# Giant Cell Tumor of the Tendon Sheath: A Rare Case of Big Toe Involvement

**DOI:** 10.7759/cureus.81601

**Published:** 2025-04-02

**Authors:** Mohamad Khalid Al Aswad, Devika Das, Marc Najjar, Maher Toulaymat

**Affiliations:** 1 Medicine, University of Sharjah, Sharjah, ARE; 2 Orthopaedics, Dr. Sulaiman Al Habib Hospital, Dubai, ARE; 3 Pathology, Dr. Sulaiman Al Habib Hospital, Dubai, ARE

**Keywords:** foot tumor, giant cell tumor, histopathology, marginal excision, orthopedic oncology, recurrence, soft tissue tumor, tendon sheath

## Abstract

Giant cell tumor of the tendon sheath (GCT-TS) is a benign yet locally aggressive proliferative lesion arising from the synovium of tendon sheaths, bursae, or joints. It most commonly affects the hands and feet, with toe involvement being particularly rare. We present a case of a 41-year-old male with a painful, firm mass on the big toe, initially suspected to be gout. Imaging studies, which included an MRI, revealed a well-defined soft tissue mass with marked cortical erosion of the first metatarsal, and histopathological examination confirmed the diagnosis of GCT-TS. The patient underwent marginal excision of the lesion, with no recurrence at follow-up. This case highlights the diagnostic challenges associated with GCT-TS in uncommon locations and highlights the utilization of MRI in assessing tumor extent and guiding preoperative planning. Despite its benign nature, the risk of recurrence necessitates complete excision and careful postoperative monitoring. The emergence of molecular therapies, such as CSF1R inhibitors, shows promise for recurrent or inoperable cases. Early recognition, precise surgical intervention, and a multidisciplinary approach in tackling this tumor are crucial for optimizing outcomes and preventing recurrence in atypical presentations of GCT-TS.

## Introduction

Giant cell tumor of the tendon sheath (GCT-TS) is a benign soft tissue tumor originating from the synovium of the tendon sheath, bursae, or joint synovium. While GCT-TS commonly affects the hand, it is rarely found in the big toe, making this case particularly unique. As described by Gouin et al. [1], GCT-TS is classified into localized and diffuse types. Localized GCT-TS, also referred to as nodular tenosynovitis, presents as a slow-growing, painless mass, while the diffuse type often mimics inflammatory or neoplastic conditions and has a higher recurrence rate. GCT-TS is known for its local aggressive behavior and is documented to be the second most common benign tumor of the hand after ganglion cysts [2]. They are known to primarily affect adults between the ages of 30 and 50, with a higher incidence in females [3]. Magnetic resonance imaging (MRI) is essential for the diagnosis and management of this tumor, as it provides detailed visualization of the tumor’s size, shape, and its relationship to the surroundings, including potential involvement of bone cortices and neurovascular structures [4]. While surgical management remains the cornerstone in the management of GCT-TS, it can be quite difficult to balance the removal of the entire tumor without significant damage to vital neighboring structures [5]. Even though this tumor is classified as benign, recurrence rates have been reported to range from 15% to 45% in some studies, with the diffuse type being more prone to recurrence compared to the localized form [6]. This report documents a rare case of GCT-TS in the big toe and integrates data from retrospective studies to provide a robust discussion on its diagnosis, management, and outcomes.

## Case presentation

A 41-year-old male presented to the hospital with a three-month history of left foot pain, swelling, and a palpable mass on the big toe. This patient also reported tenderness, numbness, and significant difficulty in walking. His medical history was significant for gout and a conservatively managed talar fracture in 2019. On examination, this patient was found to have a firm, tender 3 x 4 cm lump on the dorsal aspect of the first metatarsophalangeal (MTP) joint. The squeeze test and Tinel’s sign were positive in this patient. However, it was noted that the range of motion remained unremarkable.

**Figure 1:**
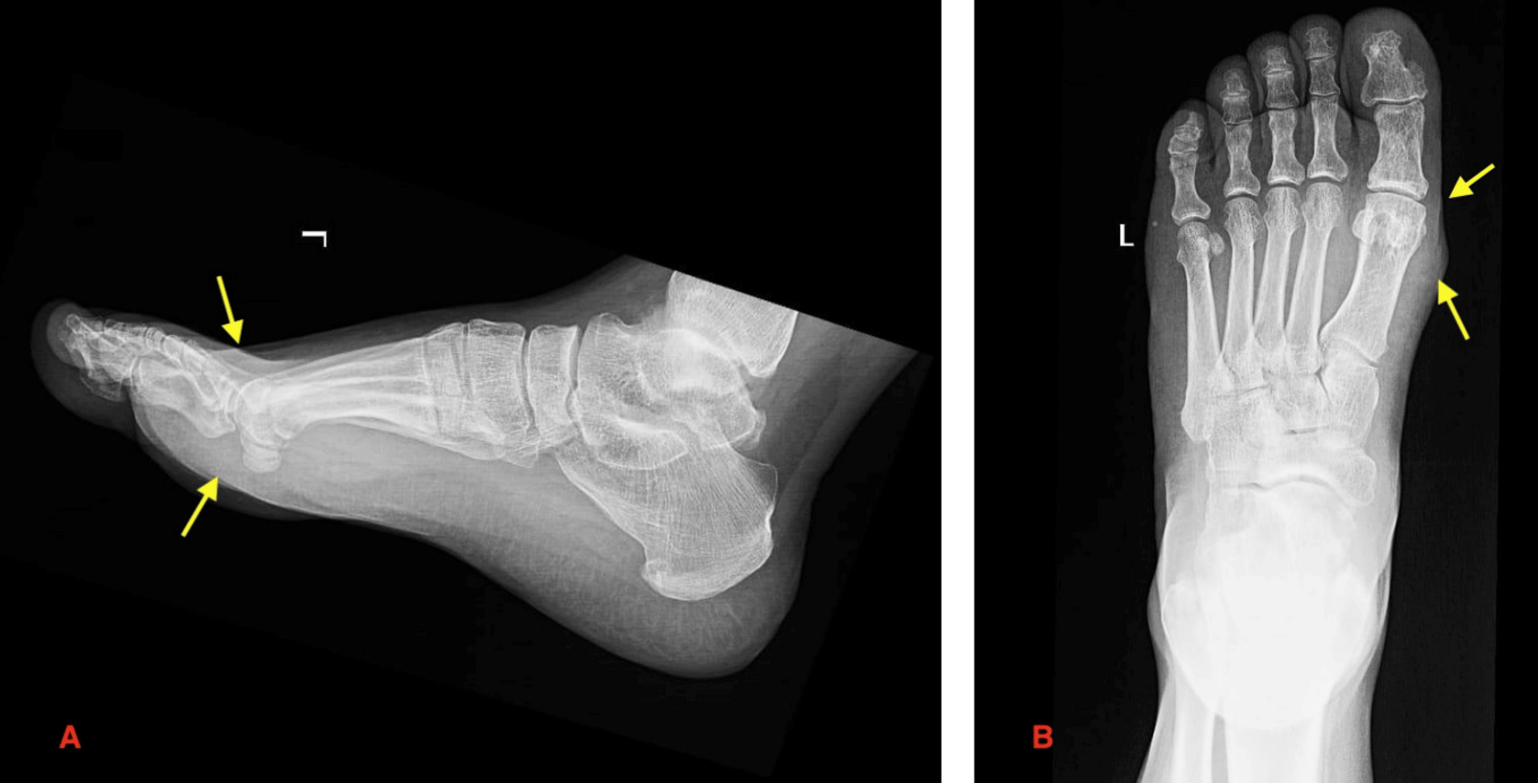
Lateral and anteroposterior (AP) X-ray views of the left foot Radiographs of the left foot in lateral (A) and AP (B) views showing evidence of thickening of the soft tissue. The surrounding osseous structures appear to be intact, with no obvious cortical erosion or periosteal reaction. Yellow arrows indicate the lesion.

**Figure 2:**
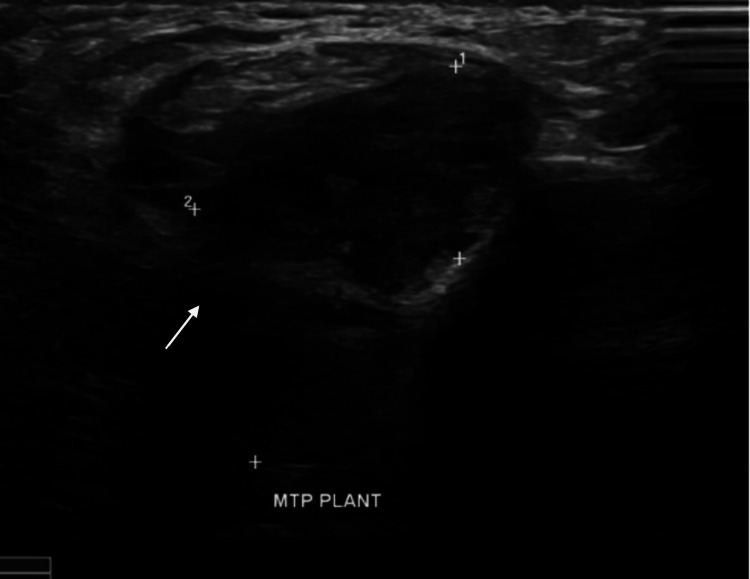
Ultrasound imaging Ultrasound scan demonstrating a hypoechoic soft tissue mass on the plantar aspect of the distal shaft and head of the first metatarsal bone. White arrow indicates the lesion.

A full radiographic evaluation was conducted, beginning with an X-ray, which showed soft tissue thickening without any significant bony destruction (Figure 1). Further assessment of this patient was conducted with an ultrasound, which revealed a hypoechoic soft tissue mass on the plantar aspect of the distal shaft and head of the first metatarsal bone (Figure 2).

**Figure 3:**
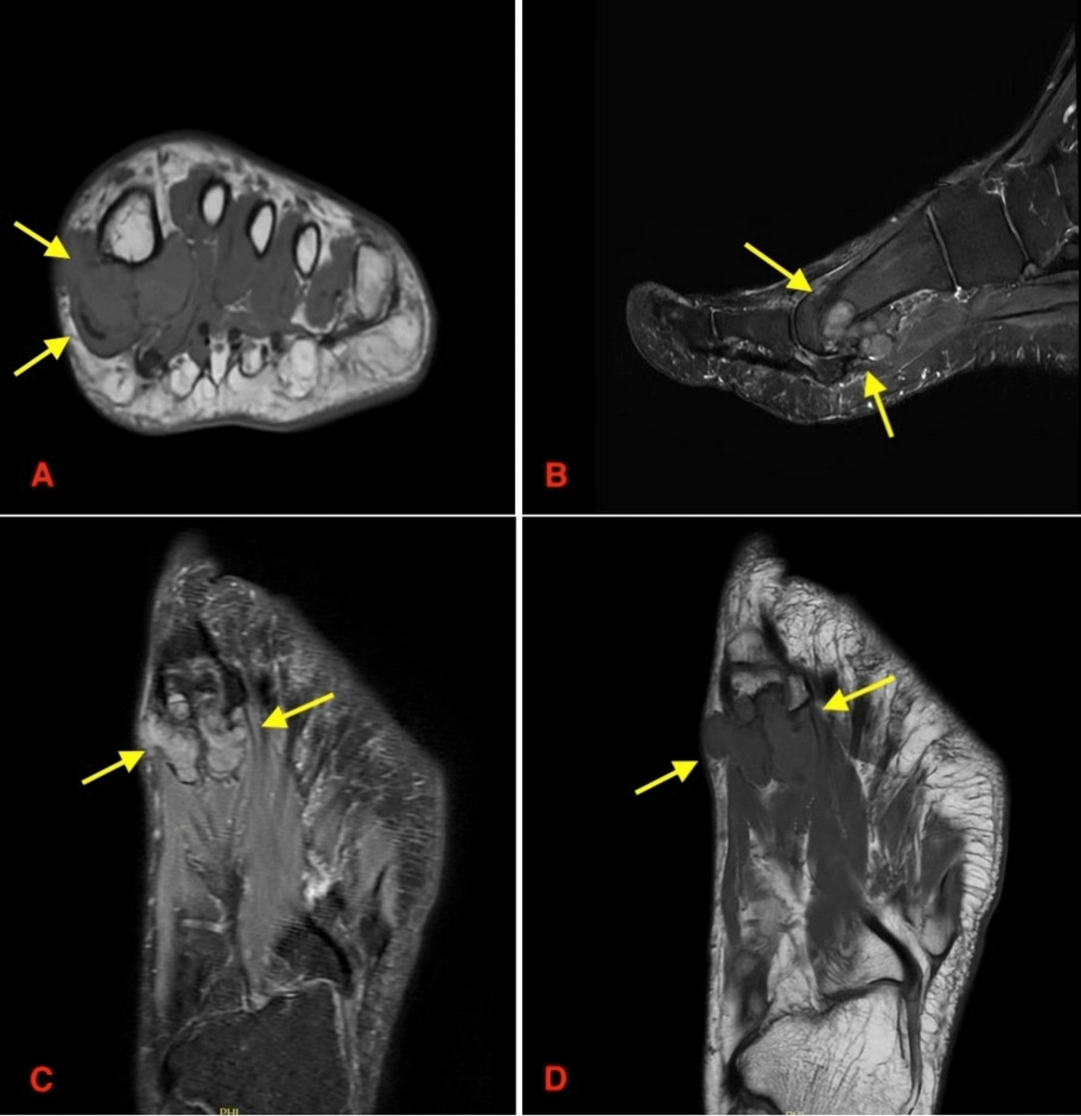
MRI findings MRI images of the left foot demonstrating a well-defined extra-articular soft tissue mass with erosion of the plantar aspect of the first metatarsal, consistent with a giant cell tumor of the tendon sheath (GCT-TS). Yellow arrows indicate the lesion. (A) Axial view showing the mass in relation to surrounding soft tissues. (B) Sagittal view showing the extent of the lesion along the plantar aspect of the first metatarsal. (C) Coronal view demonstrating the involvement of adjacent structures. (D) Coronal oblique view further showing the tumor margins and bony erosion.

**Figure 4:**
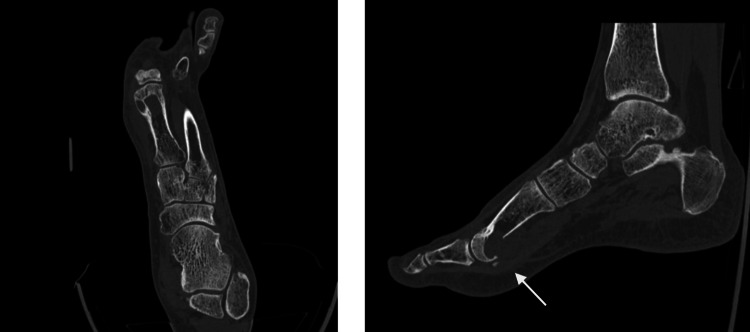
Oblique and sagittal CT views of the foot and ankle Oblique (A) and sagittal (B) CT images of the foot and ankle joint demonstrate mild erosion at the volar aspect of the distal end of the first metatarsal bone. No evidence of soft tissue calcification or uric acid deposition is observed. White arrow indicates the findings.

An MRI was then performed on this patient, which successfully provided a more detailed view, confirming a well-defined extra-articular soft tissue mass with erosion of the plantar aspect of the first metatarsal. This was consistent with a giant cell tumor of the tendon sheath (GCT-TS) (Figure 3). A CT image was then performed to rule out uric acid composition. This was important to help differentiate this lesion from tophaceous gout, given the patient's history (Figure 4).

**Figure 5:**
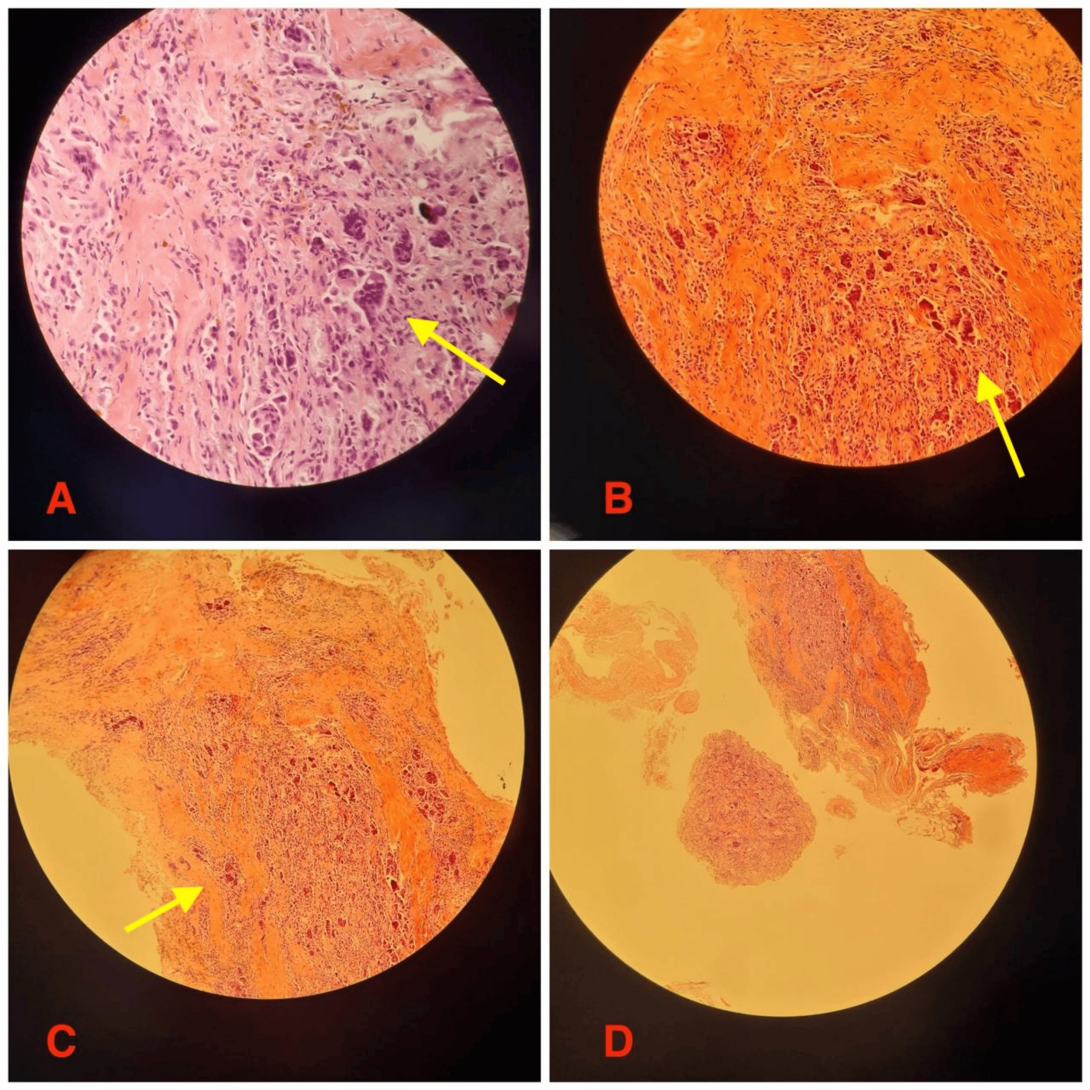
Histological features Hematoxylin and eosin (H&E) stained sections show characteristic features of GCT-TS. (A) Multinucleated osteoclast-like giant cells (dark-stained, large multinucleated cells) dispersed among mononuclear stromal cells. (B) Areas of dense cellular proliferation with spindle-shaped stromal cells and scattered giant cells. (C) Hemosiderin-laden macrophages, indicating previous hemorrhage, along with fibrotic regions. (D) Tumor architecture demonstrating mixed cellular and fibrotic components with possible bone involvement.

**Figure 6:**
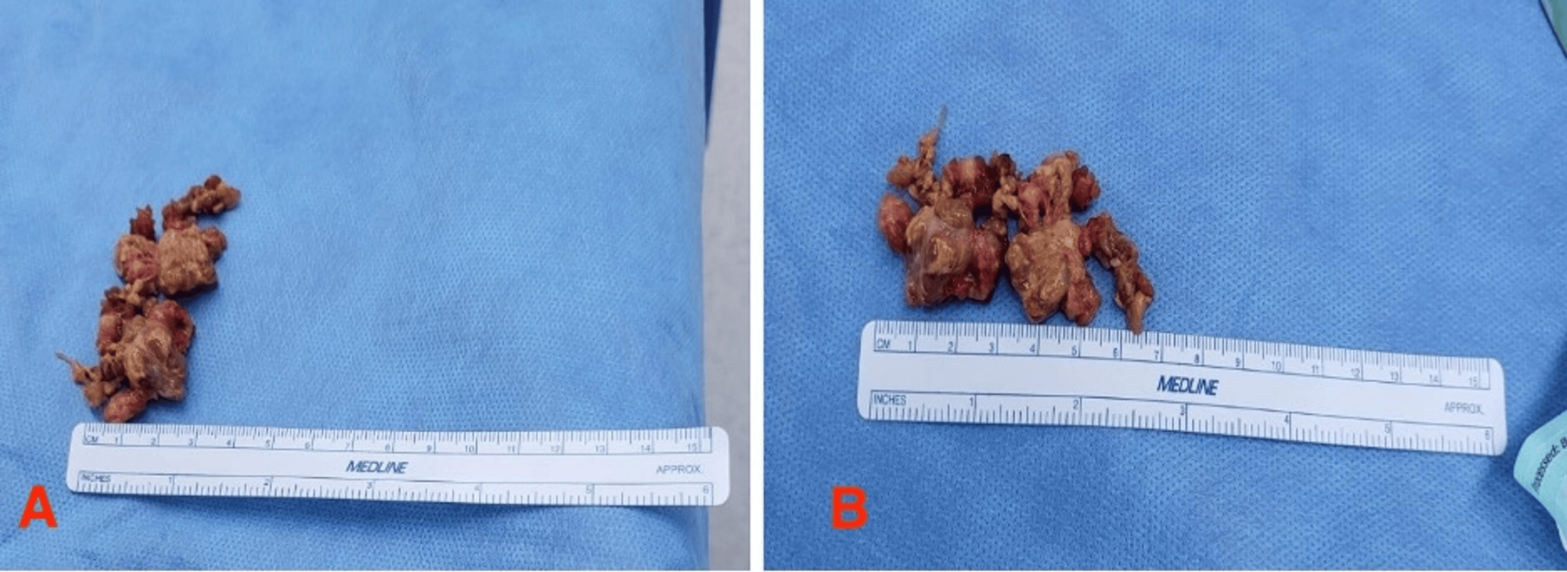
Postoperative gross examination of the excised mass (A, B) Intraoperative images showing the excised soft tissue mass. The mass is measured against a surgical ruler, confirming its size of 6.5 × 3 cm.

In order to establish a definitive diagnosis, a decision was made to perform an open biopsy. The histopathological examination revealed mononuclear cells, osteoclast-like giant cells, and hemosiderin deposits in the sample (Figure 5). Furthermore, there was no evidence of malignancy in the biopsy. The patient then underwent a marginal excision of the mass. The mass was measured to be a 6.5 x 3 cm soft tissue mass, confirmed intraoperatively, and documented with a surgical specimen ruler measurement (Figure 6). Postoperative histopathology findings successfully confirmed the diagnosis of GCT-TS.

The patient had an uneventful recovery following the surgical excision of the lesion. When the patient presented for the two-week follow-up, examination of the surgical site revealed a dry, well-healed wound. Furthermore, there were no signs of infection, and the sutures were removed. For the optimization of functional recovery, a structured physiotherapy program was planned in a goal to improve mobility and prevent future stiffness. At the four-week follow up appointment for this patient, the patient remained asymptomatic with no swelling, pain, or tenderness.

## Discussion

This case sheds light on a rare case presentation of a localization of a GCT-TS in the big toe. Given this patient’s previous history of gout, it was crucial to differentiate GCT-TS from other conditions, such as pigmented villonodular synovitis (PVNS), which shares some radiographic and clinical features. The uniqueness of the presentation reported in this patient highlights the need for meticulous diagnostic evaluation. This is especially relevant in patients with comorbid conditions that have a high chance of influencing clinical manifestations.

It is well-known that GCT-TS is the second most common cause of benign soft tissue tumors of the hand, which comprises a small fraction of all soft tissue tumors. The hand and the feet have been reported to be the most frequent locations where a GCT-TS can manifest. This observation is reiterated by studies such as those conducted by Çevik et al. [7] and Occhipinti et al. [8]. These studies found that the great majority of the studied cases were localized on the hand and less frequently on the foot. The global prevalence of toe involvement remains low, and this case adds to the growing body of literature on atypical GCT-TS presentations in less typical locations.

MRI is the optimal imaging method for the diagnosis and evaluation of the tumor, as it provides detailed information about its size, extent, and whether it has invaded nearby joints or the surrounding tenosynovial space, and this level of precision is essential for accurately characterizing the tumor, distinguishing it from other conditions, and guiding further clinical decisions [9].

Surgical excision remains the primary method of treatment for GCT-TS, with a recurrence rate ranging from 9% to 45% [10,11]. The percentages are largely dependent on the degree of resection completeness and the subtype of the tumor. In our case, the tumor was excised employing the marginal approach, which in turn led to a positive outcome with symptom resolution. With that said, recurrence of these tumors remains a great concern, particularly when the lesion is not fully excised. Studies such as those performed by Reilly et al. [10] and Kotwal et al. [11] highlight the positive correlation between achieving clear surgical margins and the reduction in the risk of recurrence. Modalities such as MRI are immensely useful for the preoperative planning of such surgeries and the identification of neighboring nerves, tendons, and bones. According to the results published by Zhang et al. [12] and Wang et al. [9], MRI findings of irregular borders and a profound bone involvement have been associated with aggressive tumor behavior and a high recurrence rate.

A significant take away from this case lies in the effect of early diagnosis, especially in areas where the resources are limited, and access to advanced imaging is not as readily available. In such cases, a delayed diagnosis can lead to unprovoked tumor progression and growth, which in turn increases the likelihood of recurrence, complicating treatment outcomes. This can be avoided through the early referral of patients suspected of having this tumor.

Research related to targeting therapies and molecular diagnostics is opening a new door for the management of GCT-TS. Furthermore, advancements in studies related to CSF1 gene mutation identifications, and its targeting with CSF1R inhibitors, such as pexidartinib, show promising results, particularly in cases of an inoperable tumor or an aggressive form [13]. As this type of research advances, while not applicable in this case due to the tumor's operable and localized nature, it may complement surgical excision in other cases.

## Conclusions

This case underscores the importance of recognizing atypical presentations of GCT-TS, particularly those that appear in uncommon locations such as the big toe, where clinical suspicion and timely imaging play a crucial role in diagnosis. While surgical excision remains the mainstay of treatment for this condition, achieving complete resection with clear margins without compromising surrounding structures is often challenging, and the risk of recurrence necessitates careful postoperative monitoring. The role of MRI in preoperative planning is crucial, as it aids in assessing tumor extent and guiding surgical decisions to optimize outcomes. Given the locally aggressive nature of GCT-TS, early intervention is critical to prevent complications and ensure favorable prognoses. Future research should focus on refining surgical techniques, improving imaging modalities for early detection, and expanding on targeted molecular therapies, such as CSF1R inhibitors, which show promise in managing inoperable or recurrent tumors. The integration of these advancements into clinical practice may further enhance patient outcomes and reduce recurrence rates.
